# Normal periocular anthropometric measurements in an Australian population

**DOI:** 10.1007/s10792-023-02669-3

**Published:** 2023-03-04

**Authors:** Khizar Rana, Mark B. Beecher, Carmelo Caltabiano, Yang Zhao, Johan Verjans, Dinesh Selva

**Affiliations:** 1 Department of Ophthalmology and Visual Sciences, South Australian Institute of Ophthalmology, University of Adelaide, North Terrace, Adelaide, SA 5000 Australia; 2 Australian Institute for Machine Learning, The University of Adelaide, Adelaide, SA 5000 Australia; 3 Royal Adelaide Hospital, Port Road, Adelaide, SA 5000 Australia

**Keywords:** Anthropometry, Australian population, Periocular, Ocular, Normative

## Abstract

**Purpose:**

To report the normative ocular and periocular anthropometric measurements in an Australian cohort and investigate how these may be affected age, gender, and ethnicity.

**Methods:**

Prospective study of patients presenting to the Royal Adelaide Hospital. Patient with orbital or eyelid disease, previous surgery, craniofacial abnormalities, pupil abnormalities, strabismus, and poor image quality was excluded. Standardised photographs were taken in a well-illuminated room. A green dot with a diameter of 24 mm was placed on the participant’s foreheads for calibration between pixels and millimetres. Ocular and periocular landmarks were segmented to calculate the periorbital measurements. Independent sample t test was used to compare male and female subjects, Pearson’s correlation was used to correlate periocular dimensions with age, and ANOVA with Bonferroni was used to compare periocular dimension between ethnic groups.

**Results:**

Seven hundred and sixty eyes from 380 participants (215 female, mean age 58 ± 18 years) were included. The mean marginal reflex distance (MRD) 1 was 3.5 mm and decreased with increasing age (*r* =  − 0.09, *p* = 0.01) and MRD 2 was 5.2 mm. Compared to Caucasians, African subjects had a significantly larger interpupillary distance and outer intercanthal distance, whereas East Asians had a significantly larger inner intercanthal distance (*p* < 0.05). The values of marginal reflex distance 2, palpebral fissure height, horizontal palpebral aperture, inner intercanthal distance, interpupillary distance and outer intercanthal distance were significantly higher in male subjects than female subjects (*p* < 0.05).

**Conclusions:**

Normative periocular dimensions may vary according to age, gender, and ethnicity. An understanding of normal periocular dimensions is important in the evaluation of orbital disease across different ethnic groups and may serve as reference points for oculoplastic surgery and industry.

## Introduction

The periocular region is a feature-rich region in the vicinity of the eyes and includes the eyelids, eyelashes, and eyebrows. Periorbital anthropometric measurements may normally vary according to age, sex or ethnicity or may be pathologically altered in craniofacial, periocular, or orbital disease [1]. Measurement of ocular and periocular structures is important in the diagnosis and follow-up of ophthalmic diseases and craniofacial abnormalities. Periocular dimensions can be assessed before and after orbital surgery to determine the impact of surgery on globe position. [2, 3]

Previous studies have evaluated the normal periocular dimensions in Indian [4], Chinese [1], Turkish [5], European [6], and Korean populations [7]. The aim of this study was to provide a normative set of periocular anthropometric measurements in an Australian cohort and investigate how these may be affected by age, gender and ethnicity.

## Methods

### Study participants

This was a prospective study conducted between September 2021 and November 2021 on patients presenting to the Ophthalmology department at the Royal Adelaide Hospital, Adelaide, Australia. Patient was included if they were older than 18 years old and consented to their participation. Patient with orbital or eyelid disease, previous surgery, craniofacial abnormalities, pupil abnormalities, strabismus, and poor image quality (e.g. blurry image) was excluded. Signed informed consent was obtained from all participants. The study was approved by the Central Adelaide Local Health Network ethics committee (CALHN reference number: 15334) and adhered to the principles of the Declaration of Helsinki.

### Image collection and analysis

Standardised facial photographs were taken in a well-illuminated room with a digital camera (Canon EOS 5D Mark II) placed at eye level approximately one metre away from the patient. The patient’s head was kept in neutral position and their eyes were in primary gaze. As a reference of scale, a green dot with a diameter of 24 mm was placed on the participant’s foreheads. The green dot was used to calibrate between pixels and millimetres.

**Fig. 1:**
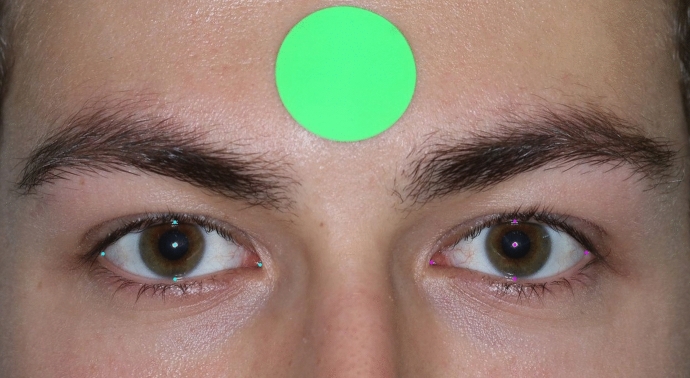
A circulator dot of 24 mm diameter was placed on the participant’s forehead. After the image was taken, the periocular landmarks were segmented with dots on Label Box

The images were uploaded onto Labelbox, which is a commercial web-based annotation tool for segmentation and classification systems [8]. Ten periocular landmarks were manually annotated including the pupillary centre, upper eyelid, lower eyelid, medial canthi, and lateral canthi for each eye by two reviewers (Fig. 1). The measurements for nineteen scans were repeated to determine the inter-rater reliability. The images were then downloaded and an open-source OpenCV library was used to calculate the distances between the periocular landmarks [9].

#### Anthropometric measurements

**Fig. 2:**
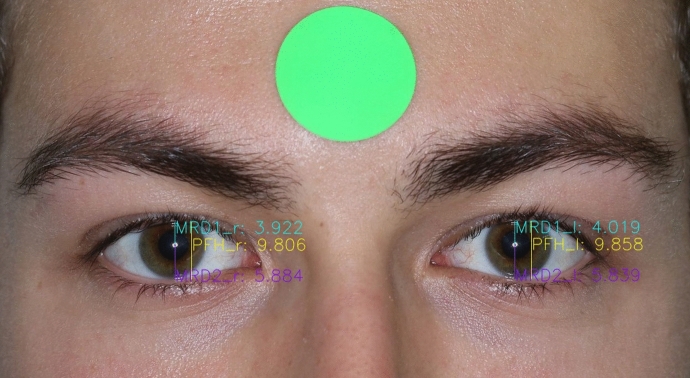
Using the segmented landmarks, the vertical distances of MRD 1, MRD 2 and PFH were calculated

**Fig. 3:**
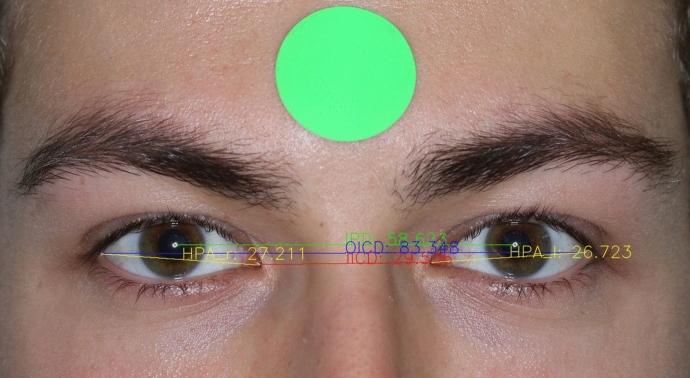
Using the segmented landmarks, the horizontal distances of IICD, IPD, OICD and HPA were calculated

Marginal reflex distance 1 (MRD1) was defined as the distance between the pupillary centre and upper eyelid margin. Marginal reflex distance 2 (MRD2) was defined as the distance between the pupillary centre and lower eyelid margin (Fig. 2). The palpebral fissure height (PFH) was measured between the superior and inferior eyelid margins and the horizontal palpebral aperture (HPA) was measured between the medial and lateral canthus. Inner intercanthal distance (IICD) was measured between the medial canthi and outer intercanthal distance (OICD) between the lateral canthi. Interpupillary distance (IPD) was measured between the centre of the pupils (Fig. 3).

### Statistical analysis

All statistical analysis was performed using Stata 13.0 (StataCorp, College Station, Texas). The mean values of periocular dimensions were presented as mean ± standard deviation. The independent samples t test was used to compare the data from male and female patients and right and left orbits. Pearson’s correlation coefficient was used to assess the correlation between age and periocular dimensions. ANOVA with Bonferroni was used to compare dimensions between age and ethnic groups. The intraclass correlation coefficient (ICC) was calculated to determine the inter-rater reliability. A P-value of less than 0.05 was considered statistically significant.

## Results

Seven hundred and sixty eyes from 380 participants (165 male, 43%) were included in the study. The overall mean age was 57.5 ± 18 years (males 56.2 years, females 58.6 years, p = 0.21). The majority of the cohort were Caucasians (316, 83%), with the other groups being East Asian (28, 7%), South Asian (27, 7%), and African (9, 2%). The mean values of periocular structures for male and female participants are presented in Table 1. The values of MRD 2, PFH, HPA, IICD, IPD and OICD were significantly greater in male subjects (p < 0.05), whereas there was no significant difference for the other parameters. The parameters from the right eyes are compared to the left eyes in Table 2.

**Table 1:** Normative periocular measurements

Measurement	Total (Mean ± SD in mm)	Male (Mean ± SD in mm)	Female (Mean ± SD in mm)	*P*-value
MRD 1	3.5 ± 0.8	3.5 ± 0.8	3.6 ± 0.8	0.80
MRD 2	5.2 ± 1.0	5.5 ± 0.9	5.0 ± 1.0	< 0.01
PFH	8.7 ± 1.3	9.0 ± 1.2	8.5 ± 1.3	< 0.01
HPA	25.6 ± 2.0	26.1 ± 2.0	25.1 ± 1.9	< 0.01
IICD	33.2 ± 3.5	33.6 ± 3.6	32.9 ± 3.4	0.048
IPD	61.3 ± 3.5	62.5 ± 3.7	60.4 ± 3.2	< 0.01
OICD	84.1 ± 4.4	85.7 ± 4.5	82.9 ± 3.9	< 0.01

*MRD* marginal reflex distance; *PFH* palpebral fissure height; *HPA* horizontal palpebral aperture; *IICD* inner intercanthal distance; *IPD* interpupillary distance; *OICD* outer intercanthal distance

**Table 2:** Comparison of right versus left eye periocular dimensions with t test

Measurement	Right eye (Mean ± SD)	Left eye (Mean ± SD)	P-value
MRD 1	3.5 ± 0.8	3.6 ± 0.8	0.01
MRD 2	5.2 ± 0.9	5.1 ± 0.96	< 0.01
PFH	8.7 ± 1.3	8.7 ± 1.3	0.51
HPA	25.6 ± 2.0	25.6 ± 2.0	0.97

*MRD* marginal reflex distance; *PFH* palpebral fissure height; *HPA* horizontal palpebral aperture

The mean values of periocular structures according to different age groups are presented in Table 3. The MRD2 and PFH of the 20–39 age group was significantly higher than each of the other age groups (p < 0.01). The HPA of the 80–99 age group and 60–79 age groups was smaller than the other age groups (*p* < 0.01). The IICD in 60–79 and 80–99 group was significantly larger than the 20–39 and 40–59 age groups (*p* < 0.01). No significant difference between groups was observed for the IPD (p > 0.05). The OICD in the 80–99 age group was significantly smaller than each of the other age groups (*p* < 0.01) (Table 4).

**Table 3:** Descriptive statistics in millimetres of the periocular structures according to age and the relationship between periocular dimension and age group by ANOVA

Measurement	20–39 (*n* = 72)	40–59 (*n* = 117)	60–79 (*n* = 163)	80–99 (*n* = 28)
MRD 1	3.65 ± 0.76	3.59 ± 0.81	3.47 ± 0.86	3.51 ± 0.92
MRD 2	5.63 ± 0.80	5.11 ± 0.98	5.04 ± 0.91	5.09 ± 1.1
PFH	9.29 ± 1.1	8.70 ± 1.3	8.52 ± 1.2	8.60 ± 1.7
HPA	26.5 ± 1.8	26.1 ± 1.6	25.2 ± 1.8	23.4 ± 2.7
IICD	32.1 ± 3.3	32.3 ± 2.9	34.0 ± 3.2	34.7 ± 5.6
IPD	60.6 ± 4.3	61.2 ± 3.3	61.9 ± 3.3	60.6 ± 3.6
OICD	84.8 ± 5.1	84.2 ± 4.0	84.2 ± 4.1	81.4 ± 4.5

*MRD* marginal reflex distance; *PFH* palpebral fissure height; *HPA* horizontal palpebral aperture; *IICD* inner intercanthal distance; *IPD* interpupillary distance; *OICD* outer intercanthal distance

**Table 4:** Association between age and periocular dimensions assessed with Pearson’s correlation coefficient

Measurement	Pearson’s correlation, r	*P*-value
MRD 1	− 0.09	0.01
MRD 2	− 0.21	< 0.01
PFH	− 0.21	< 0.01
HPA	− 0.39	< 0.01
IICD	0.26	< 0.01
IPD	0.09	0.10
OICD	− 0.14	< 0.01

*MRD* marginal reflex distance; *PFH* palpebral fissure height; *HPA* horizontal palpebral aperture; *IICD* inner intercanthal distance; *IPD* interpupillary distance; *OICD* outer intercanthal distance

A significant negative correlation was observed between age and MRD 1 (*r* = −0.09, *p* = 0.01), MRD 2 (*r* = −0.21, *p* < 0.01), PFH (*r* = −0.21, *p* < 0.01), HPA (*r* = −0.39, *p* < 0.01) and OICD (*r* = −0.14, *p* < 0.01). There was a significant positive correlation between age and IICD (*r* = 0.26, *p* < 0.01).

The mean values of periocular dimensions across different ethnic groups are presented in Table 5. The IICD in East Asians was significantly larger than Caucasians (*p* < 0.01). The IPD in African subjects was significantly larger than all other ethnic groups (*p* < 0.05). The OICD of Africans was significantly larger than Caucasians (*p* < 0.01). The ICC for inter-observer agreement was excellent with all measurements higher than 0.90.

**Table 5:** Descriptive statistics in millimetres of the periocular structures according to ethnicity and the relationship between periocular dimension and ethnic group by ANOVA

Measurement	Caucasian	East Asian	South Asian	African
MRD 1	3.56	3.47	3.47	3.28
MRD 2	5.16	5.25	5.27	5.36
PFH	8.73	8.72	8.74	8.64
HPA	25.5	25.5	26.2	26.4
IICD	32.9	35.2	33.0	35.9
IPD	61.2	61.7	61.4	65.3
OICD	83.7	85.8	85.2	88.5

*MRD* marginal reflex distance; *PFH* palpebral fissure height; *HPA* horizontal palpebral aperture; *IICD* inner intercanthal distance; *IPD* interpupillary distance; *OICD* outer intercanthal distance

## Discussion

We present the normative periocular anthropometric data in a prospectively collected cohort of 760 Australian eyes. These data may serve as a reference point for aesthetic and posttraumatic surgical interventions in a similar cohort. Age, gender, and ethnicity play an important role in the configuration of periorbital structures [4].

The MRD1 is a key measurement in the assessment of upper eyelid position. The upper eyelid is normally positioned 1–2 mm inferior to the superior limbus. Upper eyelid position can be retracted in conditions such as thyroid eye disease or reduced in a range of orbital, neurological or muscular conditions. The MRD1 is useful in classifying the degree of ptosis present and monitoring the response to treatments such as surgery. The mean MRD1 in our study was 3.5 mm, with no significant differences observed between male and female participants. The MRD 1 decreased with increasing age, likely due to age related changes within the levator muscle [10].

The palpebral fissure is the open area between the eyelids. The PFH is a sum of the MRD1 and MRD2 measurements. PFH, MRD1 and MRD2 decreased with increasing age (Table 3). Our mean PFH was 8.7 mm, with males having a larger PFH (9 mm) than females (8.5 mm). There was no significant difference between the right and left eyes, suggesting that if a difference in PFH is seen between eyes it may be an indicator of unilateral pathology. The PFH values may vary according to the population group with values of 9.7 to 9.9 mm reported in Turkish adults [11], and 8.0 to 8.2 mm in Korean adults [12]. Our study however did not reveal any significant ethnic differences in the PFH, however this may be due to type II error.

In our study, the HPA was larger in males than females and decreased with increasing age. The HPA has been reported to increase in length in the first two decades of life and then decrease with increasing age [11, 13]. The HPA may also vary according to ethnicity with higher values reported in African populations than White populations [12–14]. Our study similarly found a higher HPA in African participants than Caucasians, however, it did not reach significance likely due to the small cohort of African participants.

The IICD, OICD and IPD may be used to diagnose craniofacial anomalies. In our study, the IICD, OICD and IPD were larger in males than females. There was a significant positive correlation between age and IICD (*r* = 0.26, *p* < 0.01), and a significant negative correlation between age and OICD (*r* = −0.14, *p* < 0.01). There was no significant correlation between age and IPD. Oztürk, Yavas and Inan [11] reported a larger IICD in males than females but did not report any correlation with age.

Ethnic differences exist within the periocular dimensions of IICD, IPD and OICD. We found that African subjects had a significantly larger IPD and OICD, whereas East Asians had a significantly larger IICD. Barretto and Mathog [14] also found African subjects to have a larger IPD than White subjects. For the IICD, previous studies have suggested a mean IICD of 32 mm in a mixed cohort, similar to our study which has reported an overall mean IICD of 33 mm [15]. A Korean cohort study however found a higher IICD of 38.4 mm males and 38.2 mm in females from a Korean cohort, suggesting that Asian cohorts tend to have a larger IICD as suggested in our study as well [12].

The IPD can be used as a marker of horizontal displacement of the globe. The IPD is greater in patients with TED as compared to healthy controls [3]. The IPD can be used to monitor changes within globe position following decompression surgery for TED. The IPD decreases following medial wall decompression and balanced orbital decompression but increases with lateral wall decompression [3].

Limitations to this study include its single cohort and the cross-sectional nature. Future studies with longitudinal follow-up of patients may be able to characterise changes more closely within the periocular anatomy associated with increasing age. It may also be helpful to assess the correlation of these clinically collected periocular measurements with photographically derived variables.

This is the first Australian study to report the normative periocular dimensions in a prospectively collected cohort. These data may serve as reference points for the ophthalmic industry, clinical studies, and oculoplastic surgery.
